# Investigation of Additive Friction Stir Deposition of Inconel 718: Mechanical Performance and Microstructural Evolution

**DOI:** 10.3390/ma19122482

**Published:** 2026-06-10

**Authors:** Saeid Zavari, Selami Emanet, Huan Ding, Mahnaz Ensafi, Ehsan Bagheri, Carl Schmidt, Jeff Dulik, Shengmin Guo

**Affiliations:** 1 Department of Mechanical and Industrial Engineering, Louisiana State University, Baton Rouge, LA 70803, USA; semane1@lsu.edu (S.E.); mensaf1@lsu.edu (M.E.); ebaghe2@lsu.edu (E.B.); 2 Bechtel Plant Machinery, Inc. (BPMI), 3500 Technology Drive, Monroeville, PA 15146, USA

**Keywords:** additive friction stir deposition (AFSD), MELD, Inconel 718, nickel-based super alloys, mechanical properties, microstructural analysis

## Abstract

Additive friction stir deposition (AFSD) is a solid-state additive manufacturing process that enables the fabrication of fully dense metallic components without common fusion-related defects. Inconel 718, widely used in aerospace and energy sectors, requires high structural reliability; therefore, evaluating its response to AFSD is essential for advanced applications. This study investigates the effects of AFSD on IN718 by comparing the mechanical properties and microstructure of the as-deposited material with the feedstock condition. Tensile testing showed that the ultimate tensile strength (UTS) increased by 5% along the traverse direction, whereas elongation was reduced compared to the feedstock. In contrast, build-direction tensile specimens exhibited lower UTS and substantially reduced elongation, revealing mechanical anisotropy. Microhardness increased by 20%, consistent with substantial grain refinement from 11 µm to 3 µm due to dynamic recrystallization during deposition. X-ray diffraction (XRD) revealed no clearly detectable secondary phase formation after AFSD within the resolution limits of conventional XRD, suggesting that the increased hardness and traverse-direction strength can be partly explained by grain refinement. Elemental mapping detected oxygen-enriched Al/Ti regions at interlayer boundaries, which may contribute to the reduced build-direction ductility. Overall, AFSD refined the microstructure, enhanced hardness, and improved traverse-direction strength, while build-direction tensile testing revealed anisotropic mechanical behavior.

## 1. Introduction

Metal additive manufacturing (MAM) has become a transformative approach in modern manufacturing, offering the ability to fabricate complex and customized components directly from digital designs in a layer-by-layer manner. With advantages such as reduced material waste, shorter lead times, and greater design flexibility, MAM has found significant traction in industries such as aerospace, automotive, and energy [1,2]. Common fusion-based metal AM techniques, including Laser Powder Bed Fusion (L-PBF), Directed Energy Deposition (DED), and Electron Beam Melting (EBM), rely on high-energy heat sources to melt metallic feedstock during fabrication. While effective for a range of alloys, these processes often suffer from solidification-related defects such as porosity, hot cracking, elemental segregation, and residual stresses, primarily due to rapid cooling and thermal cycling during processing [3].

To address these challenges, solid-state additive manufacturing has emerged as a promising alternative, consolidating material through plastic deformation and interfacial bonding rather than melting [4]. Among the available solid-state AM approaches, friction-based methods have gained notable attention due to their process scalability and broad material compatibility. These include friction surface deposition additive manufacturing (FSD-AM), friction extrusion additive manufacturing (FEAM), and additive friction stir deposition (AFSD) [5]. Within them, AFSD stands out for its high deposition efficiency, suitability for large-scale production, and ability to fabricate structural components free of fusion-related defects. In AFSD, a rotating hollow tool feeds a solid consumable rod against a substrate. The combination of frictional heating and severe plastic deformation softens the material below its melting point, allowing it to be deposited in successive layers. The process achieves temperatures of approximately 50% to 90% of the material’s melting point, promoting dynamic recrystallization and strong metallurgical bonding between layers [6,7]. As a result, AFSD typically produces refined, equiaxed grain structures with reduced anisotropy, comparable to those of wrought materials. While AFSD may offer less geometric freedom than fusion-based methods, its ability to fabricate defect-free, near-net-shape parts with favorable mechanical properties has made it increasingly attractive for demanding industrial applications [7]. These advantages have generated significant interest in applying AFSD to high-performance alloys such as Inconel 718 (IN718).

IN718 is a precipitation-hardened nickel-based superalloy extensively employed in aerospace, aviation, oil and gas, and nuclear sectors owing to its exceptional tensile, fatigue, creep, and fracture strengths, combined with high-temperature oxidation resistance and chemical stability at cryogenic temperatures [8,9]. Despite its widespread use, significantly less information is available in the literature on the friction stir-based AM of nickel-based alloys. Prakash and Bauri [10] examined multiple combinations of process parameters for FSD-AM of IN718 using a rotating consumable rod/tool as the feedstock material, distinct from the hollow rotating tool configuration commonly used in AFSD. Their experimental matrix included multiple combinations of axial feed rate (10–16 mm/min), traverse speed (28–44 mm/min), and rotational speed (1000–1575 rpm). Their results showed that the optimal condition, which included a high axial feed rate (16 mm/min) and a high traverse speed (44 mm/min) with a low rotational speed (1000 rpm), produced defect-free interfaces, uniformly fine grains (2 µm), and consistent hardness (254 HV). Dilip and Janaki Ram [11] investigated friction freeform fabrication of IN718 and reported metallurgically sound deposits without defects at the layer interfaces. The process produced recrystallized fine/equiaxed grains with no formation of undesirable Laves phases. Compared to the wrought IN718, the deposits exhibited higher proof and ultimate tensile strengths, albeit with reduced tensile ductility. Santiagu et al. [12] explored the impact of post-deposition heat treatment on multi-layer Inconel 718 fabricated using the friction deposition process operated in force-control mode. To maintain surface integrity, the top of each layer was machined to remove oxidation before depositing the next. While the heat treatment did not affect grain size, it led to significant increases in hardness and ultimate tensile strength by approximately 65% and 41%, respectively. These studies demonstrate the potential of friction surfacing/deposition-based AM for IN718; however, they also indicate several process-specific challenges, including the need for surface machining between layers; sensitivity of layer geometry to axial feed, traverse speed, rotational speed, and material flow; as well as material loss through flash formation. These factors can complicate dimensional control and material efficiency in multilayer friction surfacing/deposition builds. Another solid-state route reported for IN718 is cold spray additive manufacturing (CSAM), which enables powder-based deposition without melting but commonly requires post-deposition heat treatment to improve inter-particle bonding and ductility [13]. Compared with CSAM and friction surfacing/deposition-based AM, AFSD provides a distinct bar-feedstock approach in which material is continuously fed through a rotating hollow tool and deposited under combined frictional heating, severe plastic deformation, and forging pressure.

In addition to IN718, researchers have also focused on other nickel-based superalloys, such as Inconel 625 (IN625). For example, Rivera et al. [14] reported the first study on IN625 fabricated by AFSD, showing ultrafine equiaxed grains (0.27–1 µm) from continuous dynamic recrystallization. The material exhibited higher yield and ultimate tensile strength (UTS) at both quasi-static and high strain rates, outperforming cast, wrought, and fusion-based AM counterparts. Patil et al. [15] correlated temperature and strain rate with microstructural evolution in AFSD of IN625. They achieved a 90 cm^3^/h build rate, with temperature and strain rate variations driving grain refinement from 15 µm (feedstock) to 6 µm, and 1.2 µm in inter-layer regions of the as-deposited part due to dynamic recrystallization. This refinement boosted yield strength to 753 MPa, well above feedstock material (485 MPa) and as-cast IN625 (350 MPa).

To expand the use of AFSD for fabricating high-performance components in aerospace and energy applications, it is important to understand how AFSD alters the microstructural and mechanical properties of IN718. Based on the available literature, a comprehensive study on AFSD of IN718 has not yet been published. Therefore, this study provides a detailed investigation of the microstructural evolution and mechanical performance of AFSD-processed IN718, with a direct comparison to its initial feedstock condition to evaluate the changes induced by the deposition process.

## 2. Materials and Methods

**Figure 1:**
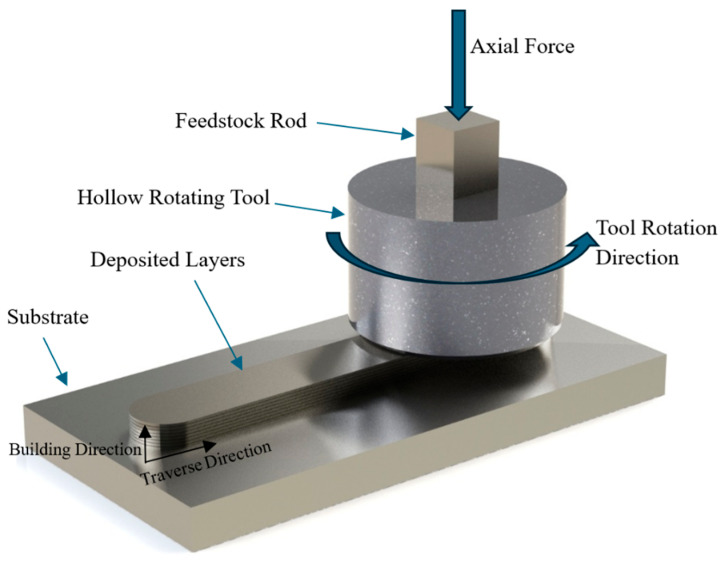
Schematic illustration of the AFSD process.

The AFSD experiments were carried out using a MELD L3 machine (MELD Manufacturing Corporation, Christiansburg, VA, USA) for layer-by-layer deposition. During the process, the rotating hollow tool and the feedstock rod generate frictional heat as the rod contacts the substrate, reducing the flow stress of the feedstock and enabling severe plastic deformation. Simultaneously, the axial pushing rod maintains constant downward motion, ensuring continuous feeding and intimate contact with the substrate or the previously deposited layer. This combination of frictional heat and axial pressure facilitates strong metallurgical bonding between layers, resulting in sound consolidation and uniform deposition. A schematic of the AFSD process, illustrating its primary components and working principles, is shown in Figure 1.

There are three primary parameters that govern the AFSD process: the spindle speed (ω), which is the rotational speed of the tool; the traverse speed (*V*), which is the linear travel speed of the tool across the substrate; and the actuator feed rate (*F*), which defines the downward advancement rate of the feedstock rod. The processing parameters used in this study were selected based on preliminary deposition trials and practical process-quality criteria. During these trials, particular attention was given to three main factors: (i) achieving continuous and stable material deposition along the tool path, (ii) maintaining the axial force below the operational limit of the MELD L3 system to avoid overloading the machine, and (iii) producing a block with no obvious macroscopic defects, particularly underfill or lack of material in the central deposition region. Accordingly, the process parameters were adjusted to minimize deposition-related issues such as excessive flash formation, underfill, and poor layer continuity, which can occur when the material flow and heat input are not properly balanced in AFSD [16]. The process parameters were finalized at ω = 500 RPM, *V* = 88.9 mm/min, and *F* = 7.62 mm/min. This parameter combination resulted in stable material flow, acceptable force levels during deposition, and a visually sound central deposition region suitable for subsequent microstructural and mechanical characterization. The correlations among these parameters can be described by the continuity equation, which defines the relationship between feedstock dimensions, traverse speed, feed rate, and the resulting layer thickness and width. This principle ensures that the amount of material entering the system equals the amount of material deposited. The relationship is expressed in Equation (1) [17]:

(1) $$Track\;Width\left(mm\right)=\frac{F\left(\frac{mm}{min}\right)\times {Area}_{bar}(mm^{2})}{V\left(\frac{mm}{min}\right)\times Layer\;Thickness(mm)},$$

**Figure 2:**
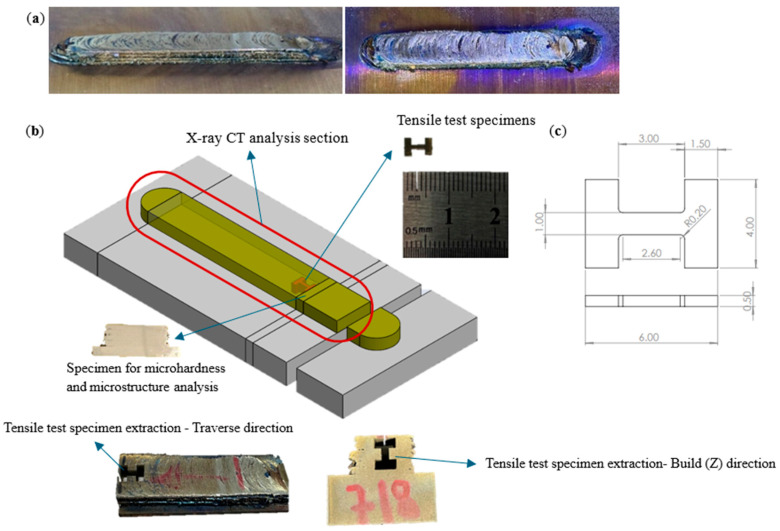
(**a**) Deposited Inconel 718 sample; (**b**) extraction locations of tensile, microhardness, and CT analysis specimens; and (**c**) dimensions of the tensile test specimen.

For the deposition, an AFSD tool with a flat surface and an outer diameter of 38.1 mm, made from W-La_2_O_3_, was employed. To enhance the bonding of the initial layer and reduce deposition forces, the substrate was preheated to 650 °C. A single IN718 rod (purchased from Continental Steel & Tube Co., Fort Lauderdale, FL, USA) in an annealed condition with a square cross-section of 9.5 mm × 9.5 mm was used as the feedstock for the AFSD process. The nominal chemical composition of the alloy is summarized in Table 1. The feedstock rod was coated with a thin layer of graphite to minimize friction and prevent jamming inside the tool. The as-deposited IN718 block measured approximately 4 mm in height (8 layers), with an intended layer thickness of 0.5 mm and an overall length of 80 mm. The block was sectioned using wire electrical discharge machining (EDM) to prepare specimens for subsequent testing, as illustrated in Figure 2.

Sub-size tensile test specimen geometry was selected for tensile samples, with a gauge length of 6 mm and a thickness of 0.5 mm (Figure 2c). Tensile test specimens were cut from the as-deposited block and the feedstock along the traverse and rolling direction, respectively. To evaluate the through-thickness tensile response, an additional AFSD block was fabricated using the same processing parameters but with a taller build height of approximately 8 mm, providing sufficient material for extracting specimens along the build direction. A minimum of three specimens was tested for each condition to ensure repeatability. Testing was carried out using an MTS 858 Mini Bionix II servohydraulic system (MTS Systems Corporation, Eden Prairie, MN, USA).

Microhardness measurements were performed in accordance with ASTM E384 [18] on both the AFSD-fabricated IN718 block and the feedstock material. For each condition, three points were measured across the cross-section to capture representative hardness values. The measurements were conducted using a CM-803 AT microhardness tester (Clark Instrument, Sun-Tec Corporation, Novi, MI, USA) with a load of 1000 gf and a dwell time of 15 s.

The samples prepared for microstructural characterization were mechanically polished using SiC sandpapers in successive grit sizes of 400, 600, 800, and 1000. This step was followed by polishing with DIAMAT polycrystalline diamond pads (PACE Technologies, Tucson, AZ, USA) of 6 μm, 3 μm, and 1 μm to achieve a mirror-like surface. Final polishing was carried out on a Pace Technologies GIGA vibratory polisher (PACE Technologies, Tucson, AZ, USA) for 24 h in pulse mode with a 35 nm silica suspension.

To characterize microstructural evolution and phase composition, multiple techniques were employed. X-ray diffraction (XRD) was carried out using a Panalytical Empyrean diffractometer (Malvern Panalytical, Almelo, the Netherlands) with monochromatic Cu Kα radiation, scanning from 10° to 120° 2θ with a step size of 0.01°. The diffraction patterns were analyzed to identify the phase constitution of the alloy. Scanning electron microscopy (SEM) was performed on a ThermoFisher Helios G5 Xe PFIB/SEM system (Thermo Fisher Scientific, Hillsboro, OR, USA) to examine the surface morphology induced by the AFSD process. Energy-dispersive spectroscopy (EDS) and electron backscatter diffraction (EBSD) mapping were also conducted on the same system to analyze chemical composition and grain structure, respectively. Grain size was calculated from EBSD data using the equivalent circular diameter method. Furthermore, X-ray micro-computed tomography (MicroCT) was performed using a Heliscan MicroCT (Mark II) system (Thermo Fisher Scientific, Hillsboro, OR, USA) equipped with a tungsten filament at 140 kV and 55 μA to investigate internal defects and porosity.

## 3. Results and Discussion

The purpose of this section is to provide a comparative evaluation of IN718 before and after processing by AFSD. For this goal, the results obtained from the feedstock material and the as-deposited AFSD block are presented and discussed in detail. The discussion focuses on the differences in mechanical properties, microhardness, and microstructural characteristics, emphasizing the role of the AFSD process in altering the alloy’s performance and microstructural integrity.

### 3.1. AFSD Data Analysis

**Figure 3:**
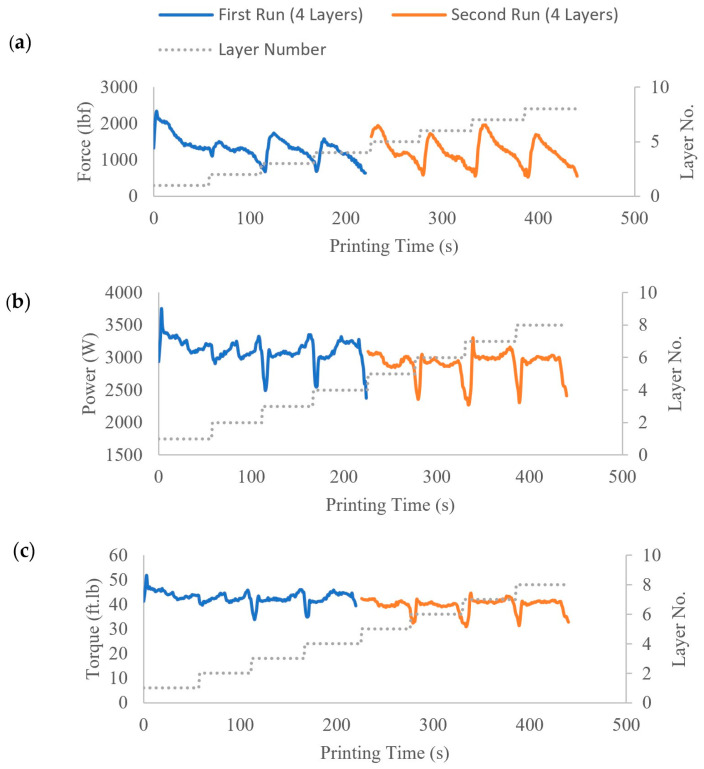
(**a**) Axial force, (**b**) power, and (**c**) torque profiles recorded during AFSD of IN718.

Before analyzing the resulting properties, it is important to examine the in-process data recorded by the MELD machine, including the variations in axial force, torque, and power during deposition. These parameters provide valuable insight into the material flow behavior and overall process consistency. Figure 3 presents the in-process data recorded by the MELD system during two deposition runs of IN718, corresponding to eight printed layers in total. Each layer shows a cyclic pattern in the axial force, characterized by an initial sharp rise followed by a gradual decrease as the tool traverses along the substrate.

This cyclic force response is associated with the material-flow behavior during AFSD of IN718. At the beginning of each layer, the feedstock rod is forced downward against the substrate by the axial actuator, causing the rod tip to deform laterally and accumulate beneath the tool as a viscoplastic deposit/flash region. As this region grows and fills the tool–substrate gap, resistance to further radial flow increases, particularly near the outer deposit region where heat loss to the surroundings is greater. This increased resistance produces the characteristic force peak observed at the start of each layer. Once the tool begins to traverse, the accumulated material is deposited onto the substrate surface, causing the axial force to decrease from its peak and gradually reach a steady state. Toward the end of each layer, the tool stops and moves upward by one layer increment before reversing direction. Continued feeding during this transition promotes temporary flash thickening, resulting in a secondary rise in axial force. Once the traverse resumes, the excess material is spread into the layer and the force decreases accordingly.

**Figure 4:**
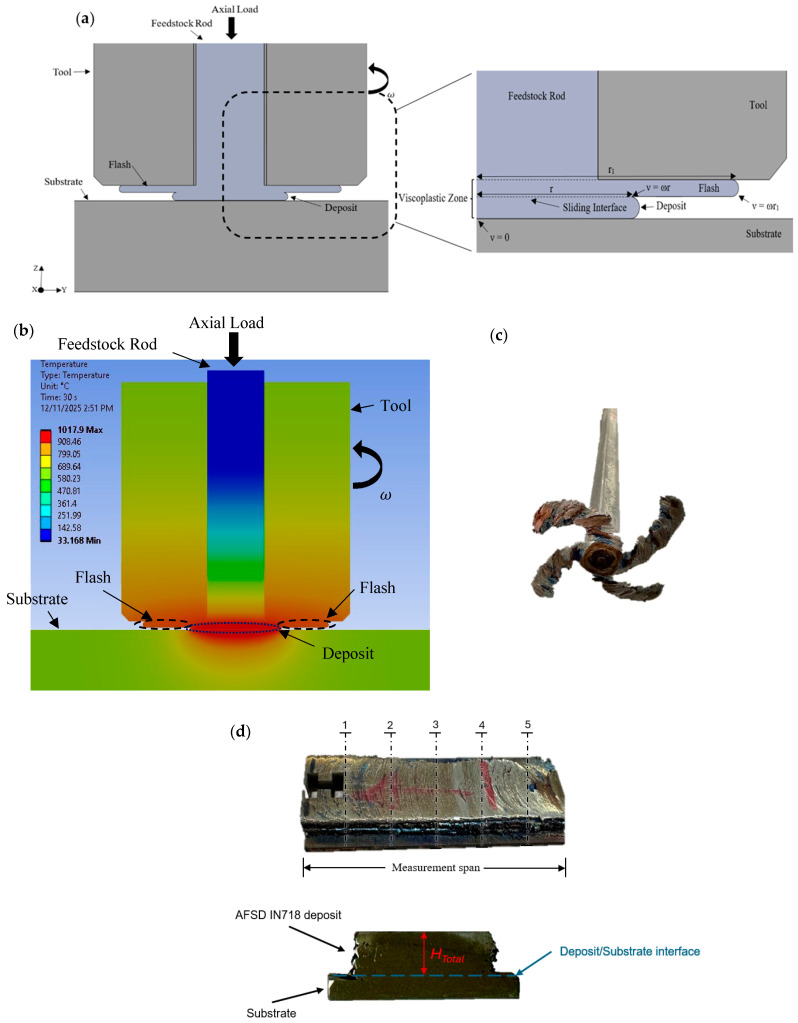
(**a**) Schematic illustration, (**b**) schematic temperature contour, (**c**) corresponding photograph showing flash formation during AFSD of Inconel 718, and (**d**) representative remaining portion of the deposited block showing the five height-measurement locations. The total build height was measured from the top surface to the deposit/substrate interface.

The material-flow behavior described above also helps identify the likely location of the dominant sliding interface during AFSD of IN718. As shown schematically in Figure 4a, the accumulated deposit/flash appears to remain connected to the feedstock rod and rotate with it during deposition. Therefore, in the present IN718 deposition, the primary sliding interface appears to develop between the rotating tool/feedstock/flash region and the stationary deposited material beneath it. The schematic temperature contour in Figure 4b supports this interpretation, showing that the highest temperatures are concentrated near this interface, where frictional heating and plastic deformation are expected to be greatest. Moving radially outward toward the flash region, the temperature decreases due to reduced contact pressure, increased free-surface exposure, and enhanced heat dissipation. As a result, the outer flash remains cooler than the central deformation region, which is consistent with the increased resistance to radial flow discussed above.

Relative to the force trend, torque and power behave differently. During the steady traverse stage, both torque and power remain nearly constant, indicating that the material flow beneath the tool is stable and sufficient for continuous deposition. However, at the end of each layer, when the tool moves upward in the Z-direction to reach the next layer height, both torque and power momentarily drop. This occurs because contact with the material temporarily decreases, reducing the resistance to rotation and consequently lowering the measured power. Once the tool re-engages with the next layer, torque and power return to their previous steady values.

Overall, this loading sequence reflects the characteristic material flow of IN718 during AFSD. As shown in Figure 4c, the feedstock rod after the eight-layer build clearly exhibits this flash interface.

This phenomenon has a direct implication on the deposition accuracy: since a portion of the plastically deformed material accumulates temporarily within the flash zone rather than being fully consolidated beneath the tool, the actual layer thickness can deviate from the programmed layer height. Consequently, achieving dimensional accuracy in AFSD of IN718 requires careful consideration of flash evolution and force control during deposition.

During the preliminary single-layer deposition trial, the target layer thickness was 0.5 mm; however, the actual retained layer height was approximately 0.2 mm lower due to flash formation. This indicated that a portion of the fed material was expelled as flash rather than being consolidated into the deposited layer. Therefore, for the subsequent eight-layer build, the initial tool/substrate clearance was set 0.2 mm larger than the target layer thickness to compensate for this height loss and obtain the desired layer thickness of 0.5 mm. The effectiveness of this compensation was evaluated by measuring the final build height at five equally spaced locations along the remaining portion of the deposited block after specimen extraction, as shown in Figure 4d, using a digital caliper. At each location, the total build height (H_Total_) was measured from the top surface of the deposited block to the deposit/substrate interface. The measured heights were 4.13, 4.17, 4.08, 4.19, and 4.04 mm, resulting in an average total build height of 4.12 ± 0.06 mm. Since the block consisted of eight deposited layers, the average actual layer height was calculated to be 0.515 ± 0.008 mm. Compared with the intended layer height of 0.5 mm, this corresponds to a deviation of approximately +3.1%. This deviation is reasonable for a deformation-based process such as AFSD.

### 3.2. Cross-Sectional and Bonding Evaluation

**Figure 5:**
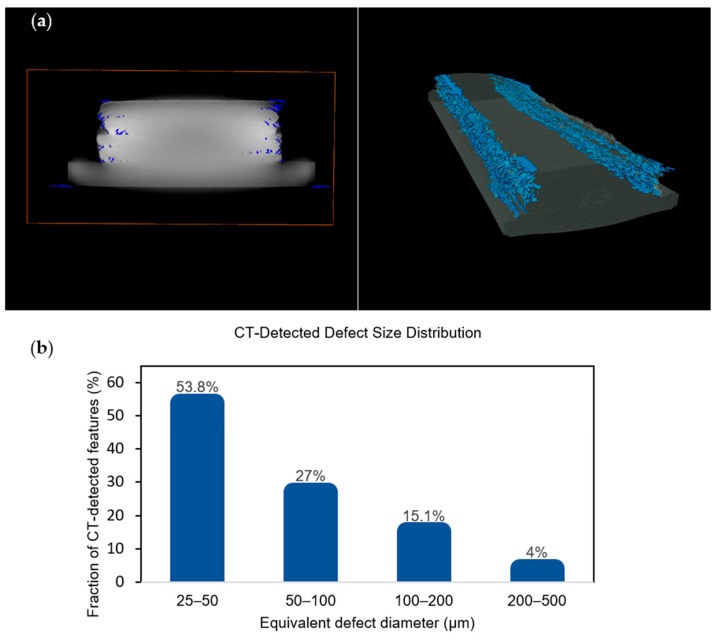
(**a**) X-ray CT scan of the AFSD-deposited Inconel 718 block showing a dense structure, with CT-detected defects highlighted in blue; (**b**) defect size distribution of the segmented CT-detected features based on equivalent diameter.

After successfully fabricating the IN718 block, the integrity of the block was first evaluated using X-ray computed tomography (CT) to ensure internal soundness in a non-destructive manner (Figure 5a). Quantitative evaluation of the CT results showed a total defect volume fraction of 1.41% within the scanned region. As shown in Figure 5b, the defect size distribution was calculated based on the equivalent diameter of the segmented CT-detected features, with mean and median equivalent diameters of 70.1 µm and 47.5 µm, respectively. Most CT-detected features were smaller than 100 µm. The CT reconstruction showed no major void network or unbonded region in the central zone, suggesting that the central portion of the as-deposited block was suitable for mechanical testing. Some defects were observed near the outer edge of the block, consistent with localized surface-adjacent disturbances typically associated with flash formation during AFSD.

**Figure 6:**
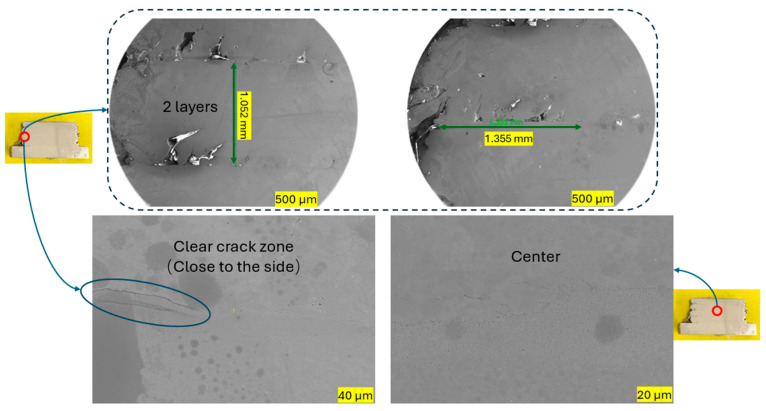
SEM images of the cross-section of the as-deposited Inconel 718 sample.

Following the CT analysis, scanning electron microscopy (SEM) was performed on cross-sectional samples to further assess layer-to-layer bonding and interface quality at a higher resolution (Figure 6). SEM images confirmed the absence of cracks or delamination in the central region of the deposit, verifying that the layers were well metallurgically bonded throughout the block. The SEM cross-section revealed a representative localized side crack near the outer edge, with a measured length of approximately 1.3 mm, consistent with the edge-region defects indicated by CT.

The deposited layer geometry was also examined. Based on the selected process parameters, each layer was expected to form with a nominal thickness of 0.5 mm. SEM cross-sections validated this expectation, showing a total thickness of 1.052 mm for two consecutive layers.

Overall, the combined CT and SEM findings confirm that AFSD produced a dense structure with uniform bonding and low porosity in the central zone of the build, which was therefore selected as the extraction region for subsequent mechanical testing and microstructural characterization.

### 3.3. Mechanical Performance and Microstructural Characterization Results

**Figure 7:**
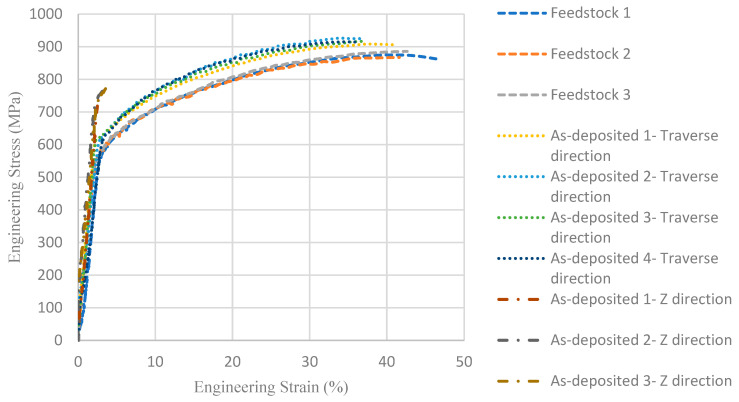
Stress–strain curves for the IN718 and as-deposited block.

**Figure 8:**
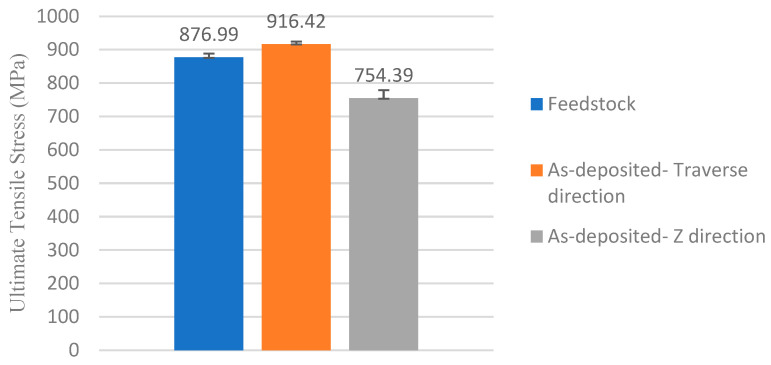
Ultimate tensile strength (UTS) of the feedstock and AFSD-processed IN718.

Figure 7 shows the tensile stress–strain curves obtained from the AFSD-processed IN718 and the feedstock material, and the corresponding tensile properties are summarized in Figure 8. The feedstock exhibited an ultimate tensile strength of 877 ± 12 MPa and elongation of 40.8 ± 0.9%. After AFSD, the UTS increased to 916 ± 9 MPa for specimens extracted along the traverse direction, while the elongation decreased to 36.2 ± 2.3%, indicating that the process improves strength but moderately limits the deformation capacity within the deposited AFSD block. In contrast, the build (Z)-direction specimens exhibited a lower UTS of 754 ± 23 MPa and a substantially reduced elongation of 3.1 ± 0.5%, indicating pronounced mechanical anisotropy between the traverse and build directions and limited plastic deformation before failure. This anisotropic response is discussed later in this section in relation to the interlayer characteristics and microstructural features observed in the as-fabricated block.

**Figure 9:**
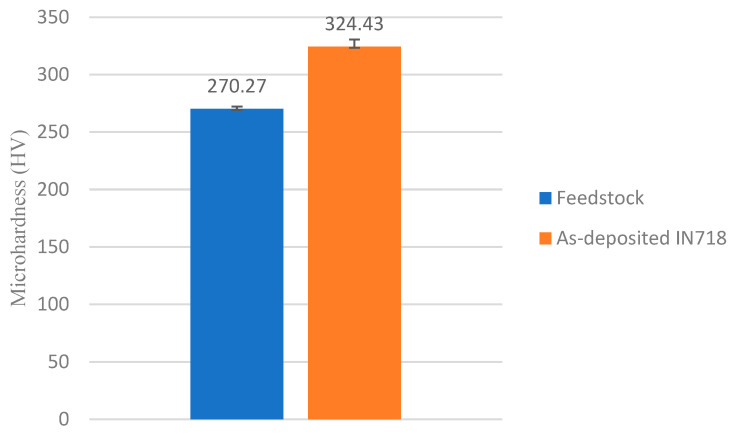
Microhardness results of the feedstock and as-deposited Inconel 718 samples.

Figure 9 presents the Vickers microhardness results for the AFSD-processed IN718 and the feedstock material. The average hardness of the feedstock was 270.3 ± 1.5 HV, whereas the as-deposited region showed an increased hardness of 324.4 ± 6.3 HV, corresponding to an improvement of approximately 20%. Together with the enhancements observed in the tensile strength along the traverse direction, these results indicate that AFSD improves the hardness and in-plane strength of IN718. To determine the microstructural origins of these property changes, detailed characterization was performed and is discussed in the following section.

**Figure 10:**
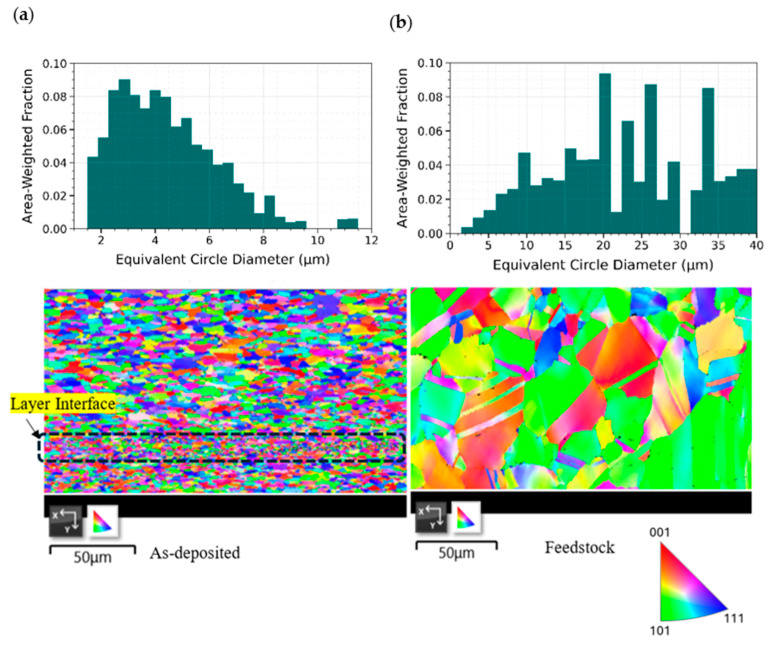
EBSD grain orientation maps and grain size distributions of the (**a**) AFSD-processed IN718 and (**b**) feedstock.

To examine the microstructural evolution induced by AFSD, electron backscatter diffraction (EBSD) analysis was conducted on polished cross-sections of both the feedstock and the as-deposited IN718. The inverse pole figure (IPF) maps in Figure 10 clearly illustrate the differences between the two conditions. The AFSD-processed region shows fine and equiaxed grains uniformly distributed throughout the deposited layer. The corresponding grain size distribution statistics (Figure 10) further confirm the substantial refinement after deposition, showing a shift in the grain population toward smaller sizes. Quantitatively, the average grain size was reduced from approximately 11 μm in the feedstock to about 3 μm in the AFSD-processed region. This grain refinement is a strong indication of continuous dynamic recrystallization promoted by severe plastic deformation and frictional heating during AFSD. Previous AFSD and FSD studies on nickel-based superalloys have shown that the most pronounced grain refinement occurs in specific regions within the deposit, particularly at interlayer interfaces [10,14,15]. EBSD-based investigations report that these regions contain significantly finer equiaxed grains compared to the surrounding material. This localized refinement has been attributed to the plastic deformation gradient imposed during the AFSD layer deposition, which leads to higher accumulated strain and promotes enhanced dynamic recrystallization at the interface regions. As a result, interlayer regions act as preferred sites for the formation of ultrafine grains, while other regions within the deposit retain relatively larger, yet still refined, equiaxed grain structures. The presence of such fine-grained regions in the as-deposited material is therefore consistent with microstructural trends reported in the AFSD literature for nickel-based alloys. This localized interfacial grain refinement may also contribute to the direction-dependent mechanical response, particularly under build-direction loading, where deformation occurs across successive layer interfaces.

**Figure 11:**
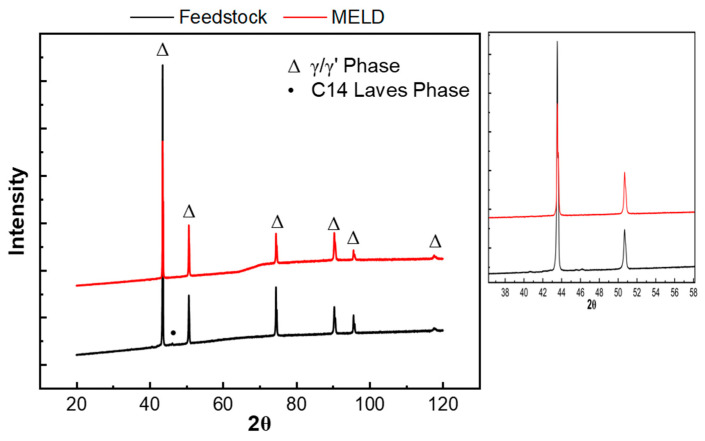
XRD patterns of the feedstock and as-deposited (MELD) Inconel 718 samples.

To determine whether the traverse-direction strength improvement and hardness increase may also be related to phase evolution, X-ray diffraction (XRD) analysis was performed on both the feedstock and the AFSD-processed material (Figure 11). The diffraction patterns of the two conditions are highly similar, with the dominant peaks corresponding to the γ matrix (FCC), which constitutes the primary ductile matrix in IN718. The as-received feedstock was supplied in the annealed condition without subsequent age hardening; therefore, the strengthening γ′ (Ni_3_(Al,Ti,Nb), L1_2_) and γ″ (Ni_3_Nb, D0_22_) precipitates are expected to be largely dissolved prior to processing [19]. As a result, no distinct diffraction peaks associated with γ′ or γ″ are expected in the feedstock. Although IN718 is strengthened through the precipitation of coherent γ′ and γ″ phases during solution treatment and aging, these precipitates typically exhibit a fine nanoscale size, high coherency with the γ matrix, and low diffraction contrast. As a result, even if limited re-precipitation were to occur locally during AFSD, these phases would not be expected to produce clearly distinguishable diffraction peaks in conventional laboratory XRD measurements [20,21,22,23]. Moreover, the total deposition time during AFSD was approximately 8 min, which is significantly shorter than the typical aging durations required for the formation and growth of γ′ and γ″ precipitates in IN718 [19,23,24]. Therefore, although transient thermal exposure during AFSD may promote limited and highly localized nucleation of these phases, the processing time is unlikely to be sufficient for the development of a substantial precipitate population detectable by conventional XRD.

A minor peak attributed to the C14-type Laves phase is detected in the feedstock, whereas no such peak is observed in the AFSD sample within the resolution of XRD. The C14-type Laves phase is a Nb-rich, brittle topologically close-packed (TCP) intermetallic phase that forms due to Nb segregation under non-equilibrium thermal conditions. Its presence is generally detrimental, as Laves phases act as preferential crack-initiation sites and locally deplete Nb from the γ matrix, thereby reducing the availability of Nb for the strengthening γ″ precipitates [20].

The absence of detectable Laves phases in the AFSD condition suggests that AFSD is unlikely to promote detrimental phase formation and may even suppress Nb-rich segregation through severe plastic deformation and repeated thermal cycling. Overall, the IN718 feedstock and the AFSD-processed IN718 share similar diffraction profiles.

**Figure 12:**
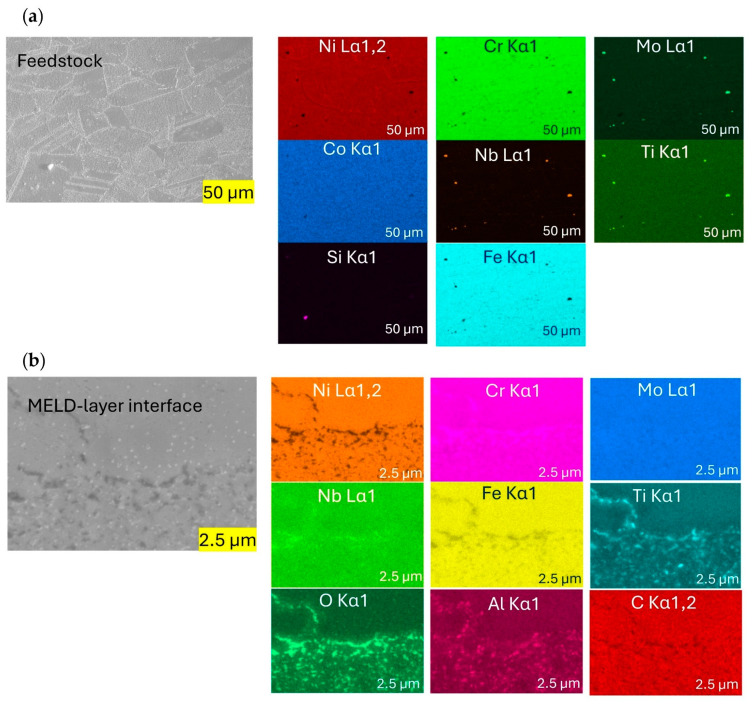
BSE and EDS mapping of the IN718 at (**a**) feedstock and (**b**) the AFSD (MELD)–layer interface.

To investigate the characteristics of the interlayer region, backscattered electron (BSE) imaging and energy-dispersive X-ray spectroscopy (EDS) mapping were performed at the interlayer region of the AFSD-processed IN718. As shown in Figure 12a, the feedstock exhibits a uniform distribution of the major alloying elements throughout the microstructure, with no detectable segregation or inclusions.

In contrast, BSE imaging of the deposited material near the layer-to-layer boundary (Figure 12b) reveals microstructural discontinuities concentrated along the interface. The corresponding elemental maps indicate oxygen enrichment at these boundaries, accompanied by localized increases in Al and Ti. The coexistence of O with Al/Ti suggests the presence of oxide films or fragmented surface oxides trapped between successive layers during deposition. These oxygen-rich inclusions may exhibit brittle fracture behavior and act as preferential crack-initiation sites [25,26], which may degrade interlayer integrity and thereby influence mechanical behavior in the building direction.

Similar oxide films have been reported in additively manufactured and solid-state bonded IN718 alloy, where the native oxide layer present on the material surface can be disrupted by severe plastic deformation during processing, leading to fragmentation of the oxide film and the formation of fine oxide particles near bonding interfaces. Depending on their size, morphology, and spatial distribution, such oxide particles may influence the mechanical response in different ways. Continuous oxide films located along interfaces can reduce metallurgical bonding and act as preferential crack-initiation sites, whereas finely fragmented oxides may interact with dislocations and potentially contribute locally to strengthening effects [27,28].

Together with the localized interfacial grain refinement observed by EBSD, the oxide-rich discontinuities at layer boundaries may increase the sensitivity of build-direction loading to interlayer features. Mechanical anisotropy between the traverse and build directions has also been reported in AFSD-fabricated alloys. For example, Lyu et al. [29] attributed early cracking and ductility deterioration under Z-direction loading to strain localization at interlayer regions, associated with alternating coarse/fine grain distributions and pre-existing strain at the interface. In the present IN718 build, the reduced elongation in the build direction may similarly reflect the combined influence of localized interfacial grain-size heterogeneity, oxide-rich discontinuities, and localized bonding imperfections. Although the build-direction specimens reached relatively high stress levels before failure, their response was largely elastic-dominated and involved very limited plastic deformation. Therefore, this apparent resistance to yielding should be interpreted cautiously and should not be considered improved build-direction plastic strength. Rather, interlayer microstructural heterogeneity and oxide-rich features may promote stress concentration and locally restrict deformation, allowing the stress to increase initially but causing early fracture once the local interlayer damage threshold is reached. In contrast, traverse-direction specimens exhibited more stable plastic deformation within the refined equiaxed grain structure.

Beyond oxide fragmentation and interlayer grain-size heterogeneity, atomic-scale defect behavior may also contribute to the interfacial bonding response during AFSD. In friction-based solid-state joining of IN718-related alloys, severe thermomechanical deformation has been shown to promote dislocation activity, dynamic recrystallization, atomic diffusion, and atomic exchange across interfaces, thereby affecting interfacial integrity and mechanical performance [30]. In addition, first-principles calculations on Ni-based γ/γ′ interfaces have shown that vacancy defects can strongly influence interfacial stability, the work of adhesion, and tensile strength by modifying charge distribution and disrupting key Ni-Al orbital hybridization [31]. Therefore, although vacancy evolution was not directly characterized in the present study, the severe plastic deformation and compressive loading imposed during AFSD may influence vacancy-type defect redistribution near interlayer regions and provide a complementary atomic-scale contribution to interfacial bonding.

In general, the mechanical strength of metallic materials is governed by several contributing factors, including (1) grain size, (2) the presence and characteristics of precipitates or secondary phases, and (3) the extent of cold work. In the present study on AFSD-processed IN718, microstructural characterization did not reveal evidence of significant secondary phases or strengthening precipitates detectable by conventional techniques. This observation is likely related to the short thermal exposure during deposition (approximately 8 min), which is considerably shorter than the aging durations typically required for the formation and growth of γ′ and γ″ precipitates in IN718. As a result, the contribution of precipitation strengthening to the measured mechanical response is expected to be limited.

In the apparent absence of pronounced precipitation effects, the mechanical behavior of the AFSD-processed material appears to be most consistently explained by microstructural refinement and deformation-related mechanisms. EBSD observations indicate extensive dynamic recrystallization within the deposited layers, suggesting that any strengthening associated with retained cold work is likely reduced during processing. Consequently, the differences in mechanical properties between the AFSD-processed IN718 and the annealed feedstock are most reasonably attributed to grain refinement induced by the AFSD process. However, contributions from other microstructural factors, such as localized interfacial grain refinement, oxide fragmentation, residual deformation structures, solute redistribution below the detection limit of XRD, or nanoscale precipitation not resolvable by conventional characterization, cannot be entirely excluded.

The AFSD-processed IN718 exhibited a marked reduction in average grain size compared to the feedstock material, leading to an increase in grain boundary density. These grain boundaries act as effective barriers to dislocation motion, thereby enhancing the material strength in accordance with the Hall–Petch relationship [32]. As grain size decreases, the resistance to dislocation glide increases, resulting in higher yield strength. The Hall–Petch relationship describing this behavior can be expressed as:

(2) $$\sigma_{y}=\sigma_{0}+kd^{-1/2},$$

where $\sigma_{y}$ is the yield strength, $\sigma_{0}$ is the overall resistance of the lattice to dislocation movement, k is a strengthening coefficient, and d is the average grain size [33]. Overall, the results suggest that grain refinement associated with dynamic recrystallization is likely a major contributor to the increased hardness and traverse-direction strength of the AFSD-processed IN718. Nevertheless, the strengthening response should be interpreted as the combined outcome of microstructural refinement and possible secondary contributions from other deformation- and interface-related features under the present processing conditions.

## 4. Conclusions

This study investigated the influence of the additive friction stir deposition process on the mechanical performance and microstructural evolution of Inconel 718, compared with its original feedstock condition. The key conclusions are summarized below:

- Assessment of the build quality:

X-ray CT and SEM analyses showed that the central region of the AFSD-processed IN718 exhibited a relatively dense and well-consolidated structure suitable for mechanical testing. Quantitative CT analysis indicated a total defect volume fraction of 1.41% within the scanned region, with most CT-detected features smaller than 100 µm. Localized cracks/defects were mainly observed near the side regions of the block.

- Mechanical properties:

AFSD improved the traverse-direction strength of IN718, increasing the UTS from 877 MPa (feedstock) to 916 MPa for the as-deposited block. Elongation in the traverse direction slightly decreased in comparison to the feedstock. In contrast, build-direction tensile specimens showed lower UTS and substantially reduced elongation, indicating anisotropic mechanical behavior and limited through-thickness ductility in the as-deposited block.

- Microhardness:

Microhardness increased by 20% after AFSD, rising from 270 HV to 324 HV. This improvement indicates enhanced resistance to plastic deformation because of the refined microstructure in the deposited layers.

- Microstructural refinement:

EBSD analysis revealed significant grain refinement from approximately 11 µm in the feedstock to about 3 µm in the as-deposited region, consistent with continuous dynamic recrystallization during AFSD. XRD patterns for both conditions were dominated by γ-matrix reflections, indicating no detectable phase evolution within the resolution limits of conventional XRD.

- Interlayer Microstructural Observations:

Oxygen-enriched Al/Ti inclusions were observed at interlayer boundaries, which may influence interlayer bonding and the overall mechanical response of the deposited material depending on their morphology and distribution. Further investigation is required to clarify their specific role.

From an application standpoint, AFSD demonstrates strong potential for fabricating high-performance Ni-based superalloys with improved hardness and strength along the traverse direction, making it attractive for structural components subjected to loading parallel to the deposited layers. However, the reduced build-direction ductility indicates anisotropic mechanical behavior and suggests that through-thickness performance remains sensitive to interlayer integrity. The observed interlayer discontinuities and oxygen-enriched Al/Ti inclusions further support the need for improved interfacial quality. Therefore, further process refinement and shielding strategies may be needed to enhance interlayer integrity for applications involving multiaxial or out-of-plane stresses.

## Figures and Tables

**Table 1:** Chemical composition of IN718 (in wt.%).

C	MN	FE	S	SI	CU	NI	NB
0.03	0.09	18.03	0.0005	0.06	0.14	53.59	5.18
CR	AL	TI	CO	MO	TA	B	P
18.25	0.53	0.98	0.17	3.02	0.1	0.002	0.008

## Data Availability

The original contributions presented in this study are included in the article. Further inquiries can be directed to the corresponding authors.
